# The role of embodied cognition in action language comprehension in L1 and L2

**DOI:** 10.1038/s41598-024-61891-w

**Published:** 2024-06-04

**Authors:** Stefana Garello, Francesca Ferroni, Vittorio Gallese, Martina Ardizzi, Valentina Cuccio

**Affiliations:** 1 Unit of Neuroscience, Department of Medicine and Surgery, University of Parma, 43126 Parma, Italy; 2 Italian Academy for Advanced Studies in America, Columbia University, New York, NY USA; 3 Department of Ancient and Modern Civilizations, University of Messina, 98168 Messina, Italy

**Keywords:** Human behaviour, Language

## Abstract

In this study we carried out a behavioral experiment comparing action language comprehension in L1 (Italian) and L2 (English). Participants were Italian native speakers who had acquired the second language late (after the age of 10). They performed semantic judgments on L1 and L2 literal, idiomatic and metaphorical action sentences after viewing a video of a hand performing an action that was related or unrelated to the verb used in the sentence. Results showed that responses to literal and metaphorical L1 sentences were faster when the action depicted was related to the verb used rather than when the action depicted was unrelated to the verb used. No differences were found for the idiomatic condition. In L2 we found that all responses to the three conditions were facilitated when the action depicted was related to the verb used. Moreover, we found that the difference between the unrelated and the related modalities was greater in L2 than in L1 for the literal and the idiomatic condition but not for the metaphorical condition. These findings are consistent with the embodied cognition hypothesis of language comprehension.

## Introduction

### Embodied Cognition and language comprehension

Following the identification of mirror neurons^1^, in the last thirty years the Embodied Cognition Hypothesis has provided considerable empirical support showing that language comprehension is closely linked to the sensory-motor system^2–7^. According to this view, words with a motor component, such as action verbs (e.g. "grasp"), are highly dependent on the reactivation of specific motor programs stored in motor cortical areas^5–8^. Consequently, reading or hearing a sentence such as “John grasps the cup" triggers the activation of hand-related areas in the motor cortex, even if no manual actions are performed^9–11^.

This mechanism operates not only in the comprehension of literal and concrete sentences, but also extends to the comprehension of figurative action sentences that convey abstract meanings through motor words (e.g. “John grasps the concept”, where the same verb used to describe physical actions is also used to convey abstract ideas – cf.^12^). In this context, studies of figurative action language using both behavioral^13^ and neuroimaging techniques, such as fMRI^14–16^ and TMS^17,18^, suggest that the degree of motor activation in the comprehension of action language forms a continuum that is modulated by various factors, including the concreteness of the stimuli. Specifically, from this standpoint, several studies have examined the interplay between language and motor system by manipulating the concreteness of action verbs at the sentence level [cf. ^19^ for a review], resulting in literal action sentences (e.g., "John grasps the cup"), metaphorical action sentences (e.g., "John grasps the concept") and idiomatic action sentences (e.g., "John grasps at straws in the crisis"). Overall, these findings showed activation of motor areas for both the literal and metaphorical conditions, but not for the idiomatic condition. These results are probably related to the fact that literal sentences and metaphorical sentences maintain a connection with the concrete and motor dimension of the verb "grasp", while idiomatic sentences are stored as a single lexical unit and have completely lost their relationship with the motor and concrete dimension of the verb^14–16^. Taken together, these findings show the graded nature of the involvement of the motor system in the comprehension of action language based on the characteristics of the stimuli.

### Embodied Cognition and second language (L2) comprehension

An alternative avenue for examining the nuanced involvement of the motor system in the processing of action language is through the lens of a second language. The extent of motor system involvement is likely to be influenced by the differences in fluency and automatization between the first language (L1) and the second language (L2). Traditionally, it is assumed that understanding a less well-spoken language (L2) involves less reliance on multimodal information and consequently a lower level of motor engagement for meaning simulation than in one's first language^20^. First, it is worth noting that the acquisition of L1 and L2 usually occurs by different processes, which may affect the extent to which they are embodied. L1 is rooted in intensive physical engagement with the environment and with people throughout the developmental period^21^. In contrast, L2 is typically acquired later in life and often facilitated by the mediating use of L1. For these reasons it has long been assumed that L2 may have a lower degree of embodiment than L1^22,23^.

However, recently, some studies have begun to demonstrate the involvement of the motor system in the comprehension of action language sentences in L2, highlighting that the extent of this involvement could be influenced by several factors and their interaction [for a review cf. ^19,24^; cf. also ^25,26^] such as culture-specific factors^27–29^, the structure of a specific language and differences in the concreteness and conventionality of stimuli^30^, the proficiency, automaticity and the level of competence of a second language^31^, the age of acquisition^32^ and the exposure to L2 environments^33,34^. Some studies comparing English and Chinese languages show interesting results in terms of the link between embodiment and L2. Su et al.^35^ and Feng et al.^30^ use priming experiments to show that embodied simulation plays a significant role in L2 comprehension and includes both literal and metaphorical sentences. Moreover, its influence appears to be directly proportional to the novelty of the metaphor. These findings are confirmed by an fMRI study by Tian et al.^36^, which shows a gradual attenuation of embodiment intensity from literal stimuli to abstract stimuli via metaphorical sentences when comparing the processing of literal, metaphorical and abstract sentences: specifically, “results overall revealed a response in motor ROIs (BA4: precentral gyrus; BA6: supplementary motor area) gradually decreasing in intensity from literal to abstract via metaphorical language in both L1 and L2” (Tian et al.^36^, 7). Moreover, some studies showed increased motor cortex activation in L2 compared to L1^24,35–37^, while other studies draw attention to the fact that despite the apparent motor activation in L2 comprehension, the embodied connections are less integrated^38^.

### Goal of the study

Despite some data seem to confirm the role of the motor system in the comprehension of action language in L2 speakers, the field of investigation is still full of contrasting evidence and some questions remain open and compelling. For instance, many of the studies cited so far confronting embodiment in a first and a second language compare literal or metaphorical sentences in languages that are both grammatically and culturally distant — such as English and Chinese or Italian and Persian^27,30,35^.

In this study, we carried out a behavioral priming experiment using action sentences with different degree of concreteness, such as literal, metaphorical and also idiomatic sentences, the latter being a category of excluded sentences by the previous experiments on the embodiment of a second language. The study was conducted by presenting the different types of sentences in Italian and English to a group of native Italian speakers with an upper intermediate proficiency of English (B2 according to the Common European Framework of Reference CEFR) that have learned the English language in homogeneous contexts (in class) after the age of ten. During the study, participants were asked to make judgments about the meaningfulness of the given action sentences after watching a video in which a hand performs an action that was related or unrelated to the verb used in the sentence.

We expected that observation of actions related to the verbs used in the sentences would facilitate the comprehension of literal and metaphorical sentences both in L1 (in line with^12,13^ and in L2 (in line with [26. 27]. Specifically, we expected an increasing difference between the unrelated and related modalities from the metaphorical sentences to the literal sentences, assuming for the latter the highest degree of facilitation in the related modality in comparison with the unrelated one. We expected no differences between the related and the unrelated modalities in the idiomatic condition in L1 since idiomatic sentences have lost their connection with the original motor component in favor of an abstract meaning^14–16^. On the contrary, we expected a significant difference between the two modalities in the idiomatic condition in English since idiomatic sentences in L2 are not as crystallized and automatically processed as in L1 but offer room for the compositional construction of their meaning depending on the speaker's experience^39^.

## Materials and methods

### Participants

Forty volunteers (21 males, mean age = 26.2 years, SD =  ± 4.1; mean years of school = 16.9 years, SD =  ± 2,3) participated in the experiment. The optimal sample size (N. 39) was estimated by means of statistical a priori sample size calculation, considering within factors (1 − ß = 0.950, α = 0.05, and effect size f = 0.250). All participants were native Italian speakers who had an English certificate attesting to their upper intermediate proficiency of English (B2). They learned the English language in homogeneous contexts (in class) after the age of ten (mean AoA = 10.9 years, SD =  ± 1.3). Their language skills were also verified administering the Cambridge English Placement Test before starting the experiment (mean score = 20.21/25, SD =  ± 1.14). In addition, they were right-handed, they had normal or corrected-to-normal vision and reported no history of neurological, psychiatric or language disorders.

Informed consent was obtained from all participants. The study was approved by the Local Ethical Committee (Comitato Etico Area Vasta Emilia Nord – AVEN; ID SIRER 5476) and was conducted in accordance with the Declaration of Helsinki (1964 and subsequent amendments).

### Materials

The stimuli consisted of 72 sentences in Italian and 72 sentences in English selected from a larger sample (see supplementary materials) and 36 videos showing actions performed with the right hand.

#### Sentences

For the experiment, 72 sentences in Italian and 72 sentences in English were used, belonging to different metalinguistic categories. Specifically, 18 literal sentences, 18 idiomatic sentences and 18 metaphorical sentences were used for each language. The literal sentences used an action verb to describe a physical and concrete action. The idiomatic sentences used the same action verb in an idiomatic way. The metaphorical sentences were predicate metaphors in which the same action verb is used metaphorically, so that no physical action was described but an abstract meaning was conveyed (see Table 1).

**Table 1:** Examples of sentences used as stimuli (see the supplementary materials for the full list of sentences).

	Literal condition	Idiomatic condition	Metaphorical condition	Nonsense
Italian	Il padre spezza il pane sul tavolo (*The father breaks bread on the table*)	Marco spezza una lancia in favore di Sergio *(Marco breaks a lance in favour of Sergio)*	La ragazza spezza il cuore del fidanzato *(The girl breaks boyfriend’s heart)*	Maria spezza il soldato di riepilogo *(Maria breaks the soldier of summary)*
English	The boy grasps the steering wheel	The leader grasps at a straws in the crisis	The professor grasps the concept	The girl grasps the cloud in the bottle ship

Idiomatic sentences are sentences whose meaning cannot be derived from the literal interpretation of individual words and which convey a cultural meaning that is closely linked to a language and society. The meaning of an idiomatic sentence is not constructed compositionally but is stored as a single semantic unit. In idiomatic action sentences (e.g. "John grasps at straws in the crisis"), the expression "grasp at straws" has lost its motor origin and its connection with the motor properties of the verb "grasp" used literally, in favor of an abstract meaning that cannot be constructed compositionally (in this case "to provide weak arguments"). On the contrary, metaphorical sentences are sentences in which one element, the target, is described in relation to another element, the vehicle of the metaphor. In our case, the meaning of "John grasps the concept", however strongly conventional it may be, is compositionally constructed, and the verb "grasp" retains a connection with the original literal meaning of the verb and the motor features associated with it.

The sentences in each condition were balanced in terms of syntactic structure (subject-verb-object), verb form (present simple, third person singular) and number of letters both in the intralinguistic comparison and in the interlinguistic comparison. They were also balanced for familiarity of the verb used, familiarity of the whole sentence, comprehensibility, concreteness, and perceived range of motion (see supplementary materials for details on construction and validation of the sentences).

In addition, we construed 18 nonsense sentences with the same action verb and the same syntactic structures of the other sentences both for Italian and for English. It should be specified that nonsense sentences served as catch trials to monitor participants’ attention, hence were not considered in data analysis.

#### Videos

For each language, 18 videos were used in which the right hand of a male actor performs the action described by the verb used in the sentences. The videos were in black and white and showed a right hand performing an action on an object. The camera was positioned 40 cm from the hand in a third-person perspective. The duration of each video was two seconds (see supplementary materials for details).

To ensure uniformity between the different conditions, the object in the video did not match the object in the literal condition. For example, the video for the verb "grasp" shows a hand grasping a cup, while the literal sentence with the verb "grasp" describes a boy grasping the steering wheel, i.e. a different action, albeit it is executed with the same effector. We adopted this approach to establish incongruence for all video-sentence pairs. Specifically, our videos show the prototypical action indicated by the verb (such as the act of grasping) but are incongruent with all sentences in the different conditions. Despite the use of the same verb, the sentences "the boy grasps the steering wheel", "the leader grasps at straw in the crisis" and “the professor grasps the concept” are incongruent with the action of grasping a cup depicted in the video. Our interest was to provide a gestural representation of the image schema of the verb by activating the image schema of "grasp" through the video, which comprises abstract and generic spatial patterns that emerge from perception and bodily interactions with the world. These patterns appear to be goal-directed and are involved in the comprehension of both literal and metaphorical sentences ^40–44^. Although the sentences convey different meanings of "grasp” under different conditions, they adhere to the same underlying image schema depicted by the video.

#### Procedure

Participants were seated 60 cm from a computer screen in a sound-attenuated room and were required to complete a semantic comprehension task procedure first for the Italian language and then for the English language. For both the languages, stimuli were written in lowercase white letters using Arial font and were presented in the center of a computer screen on a dark-grey background. All the materials were randomly presented on MatLab R2023a. Before the experiment, we provided participants with a list of English words used in the English sentences to make sure they knew their meaning.

**Figure 1:**
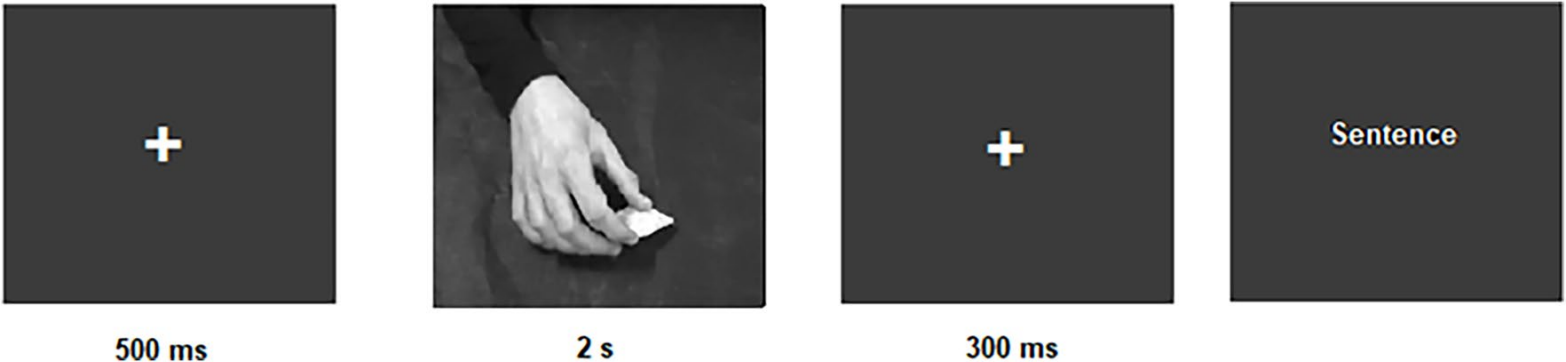
Experimental procedure. Participants were instructed to look at a fixation cross for 500 ms. Immediately after a two-second video showing a hand performing an action appeared on the screen. The video was followed by a fixation cross for 300 ms and then a sentence, related or unrelated with the action performed in the video, appeared on the screen. Participants were instructed to press a key if the sentence made sense, another key if it didn’t make sense. Reaction times and accuracy were recorded.

During the experiment, participants were instructed to look at a fixation cross for 500 ms. Immediately after, the two second video of a hand performing an action was shown, followed by a fixation cross for 300 ms. Then, a sentence appeared on the screen with a verb that was related (related modality) or unrelated (unrelated modality) to the action observed in the video both for the Italian language and the English language. All 72 sentences were presented twice: once in the related modality and once in the unrelated modality, for a total of 144 trials for the Italian language and 144 trials for the English language (see Fig. 1). Participants had to indicate whether the sentence made sense or not by pressing designated response keys (left or right) with their fingers. The lateralization of response keys was counterbalanced across participants.

Participants were encouraged to respond as quickly and accurately as possible. Once participants provided a response, the next trial began. Throughout the experiment, we recorded participants’ reaction times and accuracy. We set 10,000 ms as a maximum time of response allowed. If participants missed the response time window, the next trial was presented.

At the end of the English part of the experiment, we asked participants to translate the English sentences into Italian to make sure they had understood the meaning of the whole sentences.

### Data analysis

To investigate the efficacy of visual priming on the comprehension of sentences belonging to the three different conditions in L1 and L2, a mixed model on reaction times (RTs) was performed. We included in the analysis only accurate reaction times.

We adopted a hierarchical approach and we initially created a basic model with a single parameter. We then progressively added additional parameters to assess whether their inclusion improved the fit of the model^45^. Likelihood ratio tests as well as the Akaike Information Criterion (AIC) was used to determine whether the inclusion of main effects, interaction effects and random effects led to a model fit significantly better than the alternatives.

The model was estimated using the default type of contrast coding (treatment method). The resulting best fitting model included Modality (related, unrelated), Condition (literal, idiomatic and metaphorical) and Language (L1: Italian, L2: English) and their interactions and participant intercept as random effects.

Bonferroni post-hoc analysis was used for post-hoc comparisons among means.

The analyses were carried out with “R” software, using the following packages: *LME4 package* (mixed models^25^); *emmeans package* (post-hoc comparisons^26^); *etasquared package* (effect-size calculation^46^).

## Results

The model explained 69% of the variance in the dependent variable considering the random effect (R^2^_M_ = 0.389, R^2^_C_ = 0.689). The model revealed a significant main effect of the factors Modality, Condition, Language and, interestingly for the main purpose of the present study also the interaction Modality*Condition*Language resulted significant (see Table 2 and Table 3).

**Table 2:** Main effects and details of the model.

Main Effect	F	Num df	Den df	p	η_p_^2^
Modality	98.650	1	429	< 0.001	0.19
Condition	9.700	2	429	< 0.001	0.04
Language	446.630	1	429	< 0.001	0.01
Mod.*Cond.*Lang	2.890	2	429	0.048	0.01

**Table 3:** Fixed Effects Parameter Estimates.

				95% Confidence Interval				
Names	Effect	Estimate	SE	Lower	Upper	df	t	p
(Intercept)	(Intercept)	2.1784	0.0770	2.0276	2.32932	39.0	28.299	< 0.001
Language1	ita—eng	− 0.9272	0.0439	− 1.0132	− 0.84120	429.0	− 21.133	< 0.001
Modality1	unrel—rel	0.4358	0.0439	0.3498	0.52175	429.0	9.932	< 0.001
Condition1	idio—lit	0.2195	0.0537	0.1142	0.32479	429.0	4.085	< 0.00
Condition2	met—lit	0.0331	0.0537	− 0.0722	0.13842	429.0	0.616	0.538
Language1 ✻ Modality1	ita—eng ✻ unrel—rel	− 0.2782	0.0877	− 0.4501	− 0.10618	429.0	− 3.170	0.002
Language1 ✻ Condition1	ita—eng ✻ idio—lit	− 0.2686	0.1075	− 0.4792	− 0.05793	429.0	− 2.499	0.013
Language1 ✻ Condition2	ita—eng ✻ met—lit	− 0.0328	0.1075	− 0.2434	0.17784	429.0	− 0.305	0.760
Modality1 ✻ Condition1	unrel—rel ✻ idio—lit	− 0.2027	0.1075	− 0.4133	0.00794	429.0	− 1.886	0.060
Modality1 ✻ Condition2	unrel—rel ✻ met—lit	− 0.1316	0.1075	− 0.3422	0.07902	429.0	− 1.225	0.221
Language1 ✻ Modality1 ✻ Condition1	ita—eng ✻ unrel—rel ✻ idio—lit	− 0.2845	0.2149	− 0.7058	0.13673	429.0	− 1.324	0.186
Language1 ✻ Modality1 ✻ Condition2	ita—eng ✻ unrel—rel ✻ met—lit	0.2314	0.2149	− 0.1898	0.65268	429.0	1.077	0.282

Specifically, regarding the factor Modality, we observed faster RTs for the related modality than the unrelated modality. In relation to the factor Condition, Bonferroni post-hoc analysis revealed that the literal and metaphorical conditions were not significantly different (p = 1.000), but the literal condition was significantly different from the idiomatic condition (p < 0.001), processed the latter more slowly. There were significant differences also between the metaphorical and the idiomatic conditions (p = 0.002), with the latter, again, processed more slowly. Concerning the factor Language, the data revealed faster RTs for the Italian language than the English language (p < 0.001) (see Table 4).

**Table 4:** Mean RTs for the factors Modality (related-unrelated), Condition (literal, metaphorical, idiomatic) and Language (Italian-English).

		RT (mean, SE)	CI	p-value
Modality	Related Unrelated	1960 ms, 80 2400 ms 80	1800–2120 ms 2120–2560 ms	< 0.001
Condition	Literal Metaphorical Idiomatic	2090 ms, 83 2130 ms, 83 2310 ms, 83	1930–2260 ms 1960–2290 ms 2150–2480 ms	lit-met p = 1.000 lit-idio p < 0.001 idio-met p = 0.002
Language	Italian English	1710 ms, 80 2640 ms, 80	1550–1880 ms 2480–2800 ms	< 0.001

**Figure 2:**
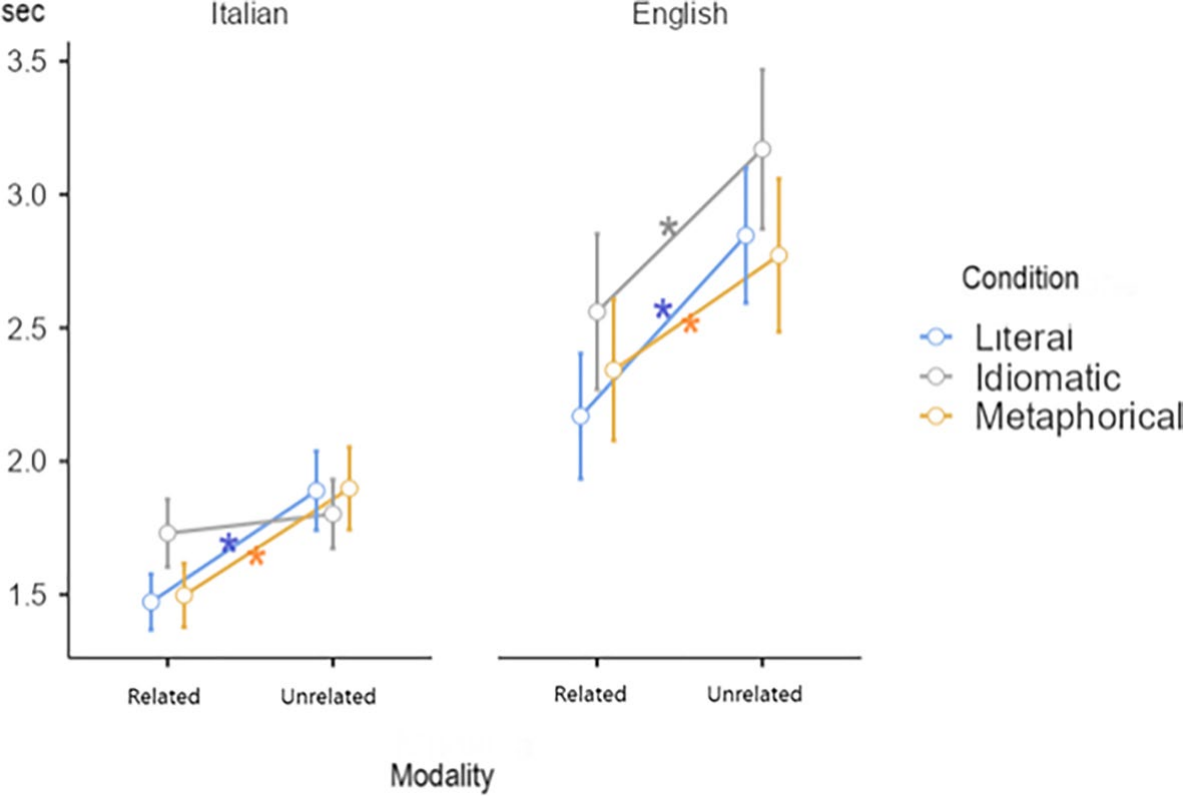
The graph shows how reaction times change depending on the condition of the sentences, both in the related modality and in the unrelated modality in Italian and in English. The asterisk [*] indicates p < 0.05; only within language significant differences are showed (see also Figure S1 in the Supplementary materials).

Finally, if we analyze the interaction Modality*Condition*Language, Bonferroni post-hoc analysis revealed that the response for the literal condition in the related modality was faster than the response for the literal condition in the unrelated modality for both Italian (p = 0.008) and English (p < 0.001). The same pattern was found also for the metaphorical condition in both the languages, observing faster RTs in the related modality rather than the unrelated modality for each language (Italian metaphorical related vs Italian metaphorical unrelated, p = 0.014; English metaphorical related vs English metaphorical unrelated, p = 0.005). On the contrary, we found a different pattern for the idiomatic condition in Italian and in English: in Italian the idiomatic related condition and the idiomatic unrelated condition did not differ significantly (p = 1.000); on the contrary, in English we found a faster RTs in the related modality rather than the unrelated modality for the idiomatic condition (p < 0.001) (see Table 5, Fig. 2).

**Table 5:** Interaction Modality*Condition*Language and the Δ value represent the subtraction of the related modality from the unrelated modality.

Language	Condition	Related RT (mean, SE)	Unrelated RT (mean, SE)	p-value	Δ_(unrelated – related)_ (mean, SE)
L1	Literal	1470 ms, 106	1890 ms, 106	0.008	417 ms, 57
L2	Literal	2170 ms, 106	2850 ms, 106	< 0.001	677 ms, 57
L1	Metaphorical	1500 ms, 106	1900 ms, 106	0.014	401 ms, 57
L2	Metaphorical	2340 ms, 106	2770 ms, 106)	0.005	430 ms, 57
L1	Idiomatic	1730 ms, 106	1800 ms, 106	1.000	72 ms, 57
L2	Idiomatic	2550 ms, 106	3170 ms, 106	< 0.001	620 ms, 57

## Discussion

In the present study, we investigated whether and in what ways action observation enhances individuals’ immediate processing of different types of action sentences that include literal, idiomatic and metaphorical sentences in both L1 (Italian language) and L2 (English language). Specifically, for both L1 and L2, participants were asked to make semantic judgements about these sentences after watching a video showing a hand performing an action that either was related or unrelated to the verb used in the sentence.

For all conditions and modalities, we found faster RTs for sentences in L1 than RTs for sentences in L2. The observed difference can be explained by the inherent automaticity of Italian as a native language, resulting in faster reading times when compared with a second language.

This result is in line with several studies showing that L1 processing is faster than L2^47–49^. Moreover, as expected, the study revealed that, regardless of the language used, responses to literal and metaphorical sentences did not differ between them in each language. These data seem to confirm various studies that have found no differences in reaction times and processing modes between literal and conventional metaphorical sentences^3,50–54^.

Indeed, for both the L1 and L2 literal and metaphorical sentences, a facilitation effect was observed in the related modality rather than the unrelated one. In these two cases in each language, congruent priming can help participants to focus on relevant motor information. The action observed in the video could therefore serve as a supportive tool in the comprehension process, activating relevant information through which the meaning of both literal and metaphorical sentences can be constructed. Participants would thus integrate the visual and motor information obtained by observing the action in the related modality with the linguistic information presented in the subsequent sentence. Conversely, in these conditions, the unrelated action could trigger information that has nothing to do with the meaning of the subsequent sentence, hence interfering with language processing.

Interestingly, we have found that the Δ value (unrelated – related modalities) in L2 is 260 ms larger compared to L1 for literal sentences: it seems, therefore, that watching action-related videos improves language comprehension, especially for the second language, at least for literal and concrete sentences. This result is consistent with the findings of Zhang et al. (2023) who show, in an electrophysiological study, that meaningful gestures provide semantic information that aids in language processing and L2 speakers derive greater benefit from these meaningful gestures compared to native speakers^55^, especially when dealing with less proficient L2 users^56^. Likewise, Ibanez et al. ^57^ and Drijvers and Ozyurek^58^ observed that in L2 speakers, unrelated meaningful gestures result in a more negative N400 response compared to their literal L1 counterparts.

Therefore, in this perspective, our study confirms that, at least for literal sentences, the difficulty of linguistic processing in the L2 is mitigated to a greater extent when multimodal cues are presented. Non-propositional entities, therefore, appear to supplement the propositional dimension when L2 speakers lack full linguistic proficiency^59^.

However, we did not find this difference between the Δ in L1 and L2 for the metaphorical condition. It is possible to hypothesize that, since literal sentences convey concrete actions that correspond to the motor cues of the action performed, showing the video of a related action might improve language comprehension for L2 speakers by providing additional contextual information that helps build sentence meaning. With metaphorical sentences, on the other hand, the situation becomes more complex. Metaphors involve abstract meanings that may have no direct or concrete counterparts in the physical world. Although the related action videos facilitate language comprehension in each of the languages taken independently, the abstractness of metaphorical sentences might make L1 and L2 comprehension more comparable under this condition. Future studies might explore whether this effect occurs systematically and examine the underlying reasons for its occurrence.

Instead, for the idiomatic condition, while in L1 no significant differences were found between the related and the unrelated modalities, in L2 we found that the RTs for the related idiomatic condition were faster than the RTs for the unrelated idiomatic condition. As already anticipated, in L1 idiomatic sentences are expressions whose meaning cannot be derived compositionally from the literal interpretation of the individual words. Instead, the meaning of an idiomatic sentence is stored as a unified semantic unit^60,61^. In idiomatic action sentences (e.g. “Marco spezza una lancia in favore di Sergio” – *Marco breaks a lance in favour of Sergio*, see Table 1), “spezzare una lancia” [to break a lance] has lost its motor origin and its association with the physical properties of the verb “spezzare” (to break) used literally. Instead, it has taken on an abstract meaning associated with the word and it cannot be constructed compositionally (as in this case “to defend”). From this point of view, the fact that we found no significant differences between the two modalities in this condition indicates that the idiomatic sentences have abandoned their motor features in favour of an abstract meaning detached from the semantic features of the action verb used.

Since the idiomatic sentences in L1 have lost their relation to the motor and concrete properties of the verb used, the observation of the action performed – both in the related and the unrelated modalities – has no effect on the comprehension of the sentence, precisely because it has no compatible or connected properties with the action presented in the video. Consequently, neither the related modality facilitates the comprehension of the idiomatic condition in L1 nor does the unrelated modality hinders it.

These results are coherent with the findings of some neuroimaging studies on L1^14–18^. In this respect, in an fMRI experiment, Aziz-Zadeh et al. ^14^ found an activation in the premotor cortex during the processing of literal and concrete action sentences but not during the processing of the more abstract idiomatic sentences. Similarly, in a TMS study, Cacciari et al.^17^, after presenting literal, idiomatic and metaphorical sentences, applied a TMS pulse over the motor area and evaluated the motor evoked potentials (MEPs) in the leg muscles. The study showed that MEPs increased with both literal and metaphorical sentences, while no comparable increase was observed with idiomatic sentences.

In an attempt to keep multiple conditions together, two more comprehensive fMRI studies also seem to support the interpretation of our data on the idiomatic condition in L1. The first experiment was conducted on native English speakers by Desai et al.^15^ and it shows an activation of motor areas only during the processing of literal and metaphorical sentences but not during the processing of idiomatic sentences. The second study, conducted on native Italian speakers by Romero Lauro et al.^16^, show similar results, finding that the level of activation of premotor areas during language comprehension depend on the degree of abstraction of the stimuli, showing therefore a general trend of decreasing motor activity (literal > metaphorical > idiomatic > abstract). Indeed, these data on L1 also confirms the findings by Garello et al.^13^.

In contrast, L2 speakers might be less familiar with the proposed idiomatic expression, hence its meaning is constructed compositionally^39^. For L2 speakers, the verb “grasp” in the idiomatic expression “grasp at straws” has not completely lost its connection to its motor origin and the physical properties of the verb “grasp” when used literally. For this reason, the observation of the action performed affects the processing and comprehension of the idiomatic sentences and thus we found a significant difference of 609 ms between the unrelated and the related modalities.

This seems to indicate that while in L1 there is a clear distinction between literal and idiomatic sentences on the one hand and metaphorical and idiomatic sentences on the other hand, which is reflected in different types of processing, in L2, speakers do not seem to make a clear distinction between idiomatic, metaphorical and literal sentences, but process all sentences as if they were literal, i.e. in a compositional way. We could hypothesize that this type of processing in L2 is probably modulated by the speaker's language level: our participants were late language learners with upper-intermediate (B2) English proficiency and a late comparable age of acquisition of the second language. The situation might be different for bilinguals or people with higher language proficiency. This issue could be an interesting point for future studies on L2, investigating also what other variables influence the processing of idiomatic expressions in L1 vs. L2 (for instance, the type of context and pattern of language use; the formal or informal way of learning L2; the daily use of a second language and so on). Moreover, for this study we used two sets of stimuli, in Italian (L1) and in English (L2), with the same group of native Italian speakers as^30,35,36^ did. We chose this approach and experimental design for two reasons: first, because of the resources and environment available to conduct the study, and second, because we felt that it was more accurate to modulate the characteristics of the two homogeneous groups of stimuli by construction than to compare two heterogeneous groups of participants by introducing variables outside our control. However, future studies could replicate this experiment by using two groups of stimuli (L1, L2) together with two groups of participants (L1, L2), thus crossing the two variables involved.

Overall, our results indicate that an integration process from different sources is used to construct the meaning of certain types of linguistic stimuli, involving perceptual and motor information in both L1 and L2 – showing, in particular, that in some cases the second language benefits more from a multimodal dimension than the first language. These findings are consistent with Embodied Cognition hypotheses^5–12^, according to which various aspects of linguistic processing are linked to our experience and our body. It can be argued that language processing is a multifaceted phenomenon involving the interaction of different and multimodal forms of representation and different sources of meaning, with embodiment being a crucial factor in the whole process^62–67^ both in the native language and in a second language. According to this perspective, language processing is not a linear process driven exclusively by linguistic representations. Rather, it seems to be a dynamic interplay of different sources of meaning in which embodiment plays a crucial role in shaping our comprehension of literal and concrete as well as abstract and metaphorical action sentences through a process of neural exploitation^5,12^.

### Supplementary Information

Supplementary Information.

## Data Availability

The dataset is available at the following link: NET 2. Other materials used during the current study are available from the corresponding author on request.
