# Supplementary material for: The role of embodied cognition in action language comprehension in L1 and L2
Source: Sci Rep. 2024 Jun 4;14:12781. doi: 10.1038/s41598-024-61891-w (PMC11150515; doi:10.1038/s41598-024-61891-w)
Supplement: Supplementary file 1 — Supplementary Information. [file 41598_2024_61891_MOESM1_ESM.docx]

**Supplementary materials**

**1. Sentences creation**

To create the sentences, we first selected 35 Italian verbs and 35 English verbs that refer to hand/arm movements. The familiarity of the verbs was rated on a Likert scale ranging from 1 (not familiar) to 7 (very familiar) by twenty Italian native speakers (10 males, mean age = 26.2 years, SD = ± 4.4; mean years of school = 16.1, SD = ± 2.2) for the Italian verbs (mean familiarity = 6.7, SD = ± 0.2) and by twenty English native speakers (9 males, mean age = 29.4 years, SD = 5.3; mean years of school = 16.3, SD = ± 1.7) for the English verbs (mean familiarity = 6.5, SD = ± 0.3). The familiarity of the verbs in Italian and that of the verbs in English did not differ significantly (p = 0.581). In both cases, participants in the validation did not participate in the main experiment.

We created three types of sentences – literal, idiomatic and metaphorical – for each action verb in both Italian and English. The sentences in each condition were balanced in terms of syntactic structure (subject-verb-object), verb form (present simple, third person singular) and number of letters both in the intralinguistic comparison and in the interlinguistic comparison. To construct the sentences, we were partly inspired by Romero Lauro *et al*. (2013) for the Italian group and Desai *et al*. (2013) for the English group.

Both in Italian and in English, the literal sentences used the action verb to describe a physical and concrete action. The idiomatic sentences used the same action verb in an idiomatic way. The metaphorical sentences were predicate metaphors in which the same action verb is used metaphorically, so that no physical action was described but an abstract meaning was conveyed. In addition, these metaphorical sentences were conventional metaphors, namely metaphors rooted in our everyday language (see Lakoff & Johnson, 1980). To measure the conventionality of the metaphors we used frequency and familiarity as indicators, considering “conventional” metaphors with a mean frequency ranging from 0.100 to 0.300 occurrences per million tokens in each corpus. Through SketchEngine we measured how often the verb and the object occur together in the Italian Web Corpus (itTenTen) for Italian sentences and in the English Web Corpus (enTenTen) for the English sentences. We ensured that there was no difference in the frequency of occurrence of verb and object in metaphorical sentences in Italian and English (mean frequency ita = 0.152, SE = ± 0.02; mean frequency eng = 0.371, SE = ± 0.2; p = 0.285).

In this way 37 sentences were created for each condition in both languages for a total of 111 sentences for the Italian group and 111 sentences for the English group.

**2. Sentences validation**

Once created, these sentences were validated by the same groups previously mentioned of twenty Italian native speakers for the Italian sentences and twenty English native speakers for the English sentences. In both cases, they were asked to rate them on a Likert scale from 1 (low) to 7 (high) for familiarity, comprehensibility, concreteness, and perceived range of motion. In the questionnaire, we carefully defined these parameters and the required response mode to minimize the intercultural differences between Italian and English native speakers for each group of sentences. Furthermore, for figurative sentences participants were also asked to write down the meaning of the verb in the sentence to ensure clarity and transparency of the figurative meaning.

In both the languages, a series of Tukey or Kruskall Wallis tests (based on the normality and variance of the data) revealed that the selected sentences differed according to the degree of comprehensibility (p < 0.001), concreteness (p < 0.001) and perceived range of motion (p < 0.001) but not for their degree of familiarity (p = 0.070) (see Tables 1, 2).

Specifically, regarding the Italian language, post-hoc analysis performed on familiarity revealed that, as expected, the literal condition was not significantly different from the idiomatic (p = 0.171) and metaphorical (p = 0.151) conditions and there were not significant differences either between the idiomatic and the metaphorical conditions (p = 0.991).

Regarding comprehensibility, the literal condition was the most comprehensible condition and significantly different from the idiomatic condition (p = 0.001) and the metaphorical condition (p < 0.001), while the idiomatic and the metaphorical conditions did not differ significantly between them (p = 0.897).

Regarding concreteness, as expected, the literal condition differed significantly from the idiomatic and metaphorical conditions (both p < 0.001) and also the idiomatic and the metaphorical conditions differed between them (p = 0.009).

With respect to the perceived range of motion, the literal condition differed significantly from the idiomatic and the metaphorical conditions (both p < 0.001) while the idiomatic and the metaphorical conditions did not differ significantly between them (p = 0.298).

Likewise, in the English language post-hoc analysis performed on familiarity revealed that the literal condition was not significantly different from the idiomatic (p = 0.590) and the metaphorical (p = 0.449) conditions and neither the idiomatic nor the metaphorical conditions differed between them (p = 0.999).

Regarding the degree of comprehensibility, also in this case the literal condition was the most comprehensible and differed significantly both by the idiomatic (p = 0.009) and the metaphorical (p = 0.034) conditions, while the idiomatic and the metaphorical conditions did not differ between them (p = 0.989).

As expected, with reference to concreteness, the literal condition differed significantly from the idiomatic (p < 0.001) and the metaphorical (p < 0.001) conditions and the idiomatic and the metaphorical conditions also differed between them (p = 0.059).

Finally, considering the perceived range of motion, the literal condition differed again from both the idiomatic (p < 0.001) and the metaphorical (p < 0.001) conditions but there were no differences between the idiomatic and the metaphorical conditions (p = 1.000).

After analyzing the data resulting from the validation of stimuli in each language, we applied a series of t-tests between Italian and English for each parameter and under each condition to verify if there were significant differences between the stimuli in the two languages.

Therefore, regarding the familiarity parameter, we applied a t-test between the Italian literal condition and the English literal condition and found no significant differences (p = 0.266); we also found no significant differences between the idiomatic condition in Italian and the one in English (p = 0.495) and, finally, no significant differences were observed between the Italian metaphorical condition and the English metaphorical condition (p = 0.459). Similarly, for concreteness, we found no significant differences between the two languages, neither for the literal condition (p = 0.164), nor for the idiomatic condition (p = 0.498), nor for the metaphorical condition (p = 0.994). Finally, we found no significant differences between the two languages in reference to comprehensibility (literal condition: p = 0.157; idiomatic condition: p = 0.097; metaphorical condition; p = 0.325) and to the perceived range of motion (literal condition: p = 0.584; idiomatic condition: p = 0.313; metaphorical condition: p = 0.557).

|  | **Literal Condition**  **(mean, SE)** | **Idiomatic Condition**  **(mean, SE)** | **Metaphorical Condition**  **(mean, SE)** | **p-value** |
| --- | --- | --- | --- | --- |
| **Familiarity** | 6.260, 0.05 | 6.050, 0.101 | 6.030, 0.070 | p = 0.070 |
| **Comprehensibility** | 5.490, 0.04 | 5.060, 0.090 | 5.000, 0.080 | p < 0.001 |
| **Concreteness** | 6.110, 0.070 | 4.180, 0.165 | 3.510, 0.126 | p < 0.001 |
| **Perceived range of motion** | 5.040, 0.196 | 3.320, 0.216 | 2.930, 0.117 | p < 0.001 |

Table 1. Italian scores averaged across all psycholinguistic dimensions for the sentences used in the experiment.

|  | **Literal Condition**  **(mean, SE)** | **Idiomatic Condition**  **(mean, SE)** | **Metaphorical Condition**  **(mean, SE)** | **p-value** |
| --- | --- | --- | --- | --- |
| **Familiarity** | 6.160, 0.07 | 5.960, 0.070 | 5.930, 0.070 | p = 0.070 |
| **Comprehensibility** | 5.380, 0.06 | 4.770, 0.137 | 4.860, 0.119 | p < 0.001 |
| **Concreteness** | 5.910, 0.121 | 4.330, 0.143 | 3.920, 0.090 | p < 0.001 |
| **Perceived range of motion** | 4.920, 0.109 | 3.080, 0.080 | 3.060, 0.193 | p < 0.001 |

Table 2. English scores averaged across all psycholinguistic dimensions for the sentences used in the experiment.

At the end of the validation process we selected 18 sentences for each condition both in Italian and in English, balancing the previously controlled attributes, for a total of 54 sentences for the Italian group and 54 sentences for the English group.

In addition, we construed 18 nonsense sentences with the same action verb and the same syntactic structures both for Italian and for English. We ensured that these sentences did not make sense by validating them with the same group of twenty Italian and twenty English native speakers. No-sense sentences were only useful for completing the experimental design and served as catch trials to monitor participants’ attention, hence were not considered in data analysis.

**LIST OF SENTENCES**

| **ITALIAN LITERAL CONDITION** | **ITALIAN IDIOMATIC CONDITION** | **ITALIAN METAPHORICAL CONDITION** |
| --- | --- | --- |
| La giovane ragazza apre la finestra verde | Carla apre gli occhi all'amica ingannata | Il capo apre nuove possibilità a Luca |
| Il ragazzo chiude la porta della stanza | Il vigile chiude un occhio sul reato | Il cantante chiude l'intensa giornata |
| Il bambino si gratta le punture di zanzara | Mario si gratta la pancia tutto il giorno | La ragazza gratta i soldi del padre |
| Il bambino taglia il foglio di carta | Il ladro taglia la corda improvvisamente | Il leader taglia le relazioni con l’India |
| Lo studente butta gli appunti di inglese | Luca butta là un'idea per la serata | Andrea butta via i soldi del padre |
| Il padre spezza il pane caldo sul tavolo | Marco spezza una lancia a favore di Sergio | La ragazza spezza il cuore del fidanzato |
| Il giardiniere pianta rose in giardino | L’ospite pianta le tende dai vicini | Il ragazzo pianta la sua fidanzata |
| La mamma tocca la spalla del bambino | Il politico tocca il fondo molte volte | Lo scrittore tocca i cuori con le parole |
| Il padre tira il portone dietro di sé | Lo studente tira la cinghia a fine mese | Lo studente tira il fiato dopo l'esame |
| La sarta cuce il vestito perla recita | Mario cuce i panni addosso al candidato | Maria cuce le ferite dell'amore violento |
| L’operaio alza i mobili per trasportarli | Il bambino alza gli occhi al cielo | Marco alza la temperatura della casa |
| L’impiegato batte le lenzuola pulite | Il soldato russo batte in ritirata | Il giocatore batte il record di gol segnati |
| La ragazza lava ogni mattina i capelli | Il maestro si lava le mani del problema | Il politico si lava la coscienza |
| Maria segna i punti del giocatore | Il politico segna il passo di una nuova era | Il leader segna l'avvio di una rivolta |
| Il cuoco sparge il sale sulle patate cotte | Il cantante sparge la voce sul concerto | Il giornalista sparge notizie false |
| Maria stringe la cintura dei pantaloni | Il ragazzo stringe i denti e resiste | Il giovane ragazzo stringe amicizie |
| Il musicista tende le corde del violino | La mamma tende una mano al figlio | Il maestro tende trappole allo studente |
| Lo studente volta le pagine del libro | Camilla volta pagina nella sua vita | La mamma volta la situazione in casa |

| **ENGLISH LITERAL CONDITION** | **ENGLISH IDIOMATIC CONDITION** | **ENGLISH METAPHORICAL CONDITION** |
| --- | --- | --- |
| The boy grasps the steering wheel | The leader grasps at a straws in the crisis | The professor grasps the concept |
| The craftsman lifts the pebbles | The leader lifts the veil on the government | The young priest lifts his spirit |
| The dad picks up the cake for his child | The student picks holes in the argument | The teacher picks the correct words |
| The new player catches every ball | The actress catches on fire again | Sam catches the crowd’s attention |
| The student raises his hand to speak | The store owner suddenly raises the bar | The student raises many questions |
| The professor knocks on the door | The singer knocks my sock off | The migrant knocks on UE’s door |
| The chef squeezes lemon over the chips | The teacher puts the squeeze on students | Sam squeezes money out of his father |
| The boy tosses the ball into the stand | The child tosses his cookies after lunch | The researcher tosses new ideas |
| The boy pours water on his noodles | The girl pours money down the drain | The leades pours resources into the war |
| The strong man tears off the door | The boy tears the girl’s heart out | The fighter tears out the guts of the enemy |
| Samuel wipes the blackboard clean | The boss wipes Mary’s slate clean | The clergyman wipes away his sin |
| The grandmother cuts the onions | The hard worker cuts the mustard | The new president cuts the staff |
| The worker tightens the bolts hard | The new president tightens its belt | The major tightens the regulations |
| The worker loosens the bolts quickly | Mary loosens her tongue with vodka | The major loosens the anticovid rules |
| He pulls the rope around the mast | The boss pulls the plug on the programme | The politician pulls his support |
| The woman tickles gently her child | The actor nicely tickles my fancy | The barman tickles their senses |
| The leader shakes the major’s hand | He shakes the party to the foundations | The singer shakes their emotions |
| Daniel hammers a nail in the wall | The teacher hammers the lesson home | The brave soldier hammers the enemy |

**2. Video validation**

We recorded a series of videos in which a male actor’s right hand executed the action described in each selected action verb for both Italian and English languages. From the overall set of videos, for each language 18 videos were selected by asking to another group of twenty Italian native speakers for the Italian verbs (10 males, mean age = 25.9 years, SD ± 3.9; mean years of school = 16.4 years, SD = ± 1.7) and another group of twenty English native speakers for the English verbs (10 males, mean age = 27.9 years, SD = ± 4.2, mean years of school = 16.6 years, SD = ± 1.8) to choose out of four options which action was performed in the video. Of the proposed options in the validation, one contained the correct answer, one contained an incorrect verb, one consisted of a synonym of the corrected verb and the last one contained “none of above”. For both Italian and English languages we selected only videos with an accuracy rate greater than 80%, without significative differences between the two groups (p = 0.740). Indeed, the two groups that validated the videos did not take part to the main experiment.

**2.1. Selected Italian videos.**

| **Action** | **Mean Accuracy** | **Frame taken from the video** |
| --- | --- | --- |
| Alzare  (Raise) | 80% | **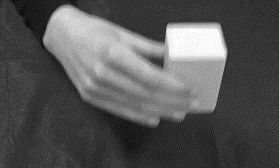** |
| Aprire  (Open) | 100% | 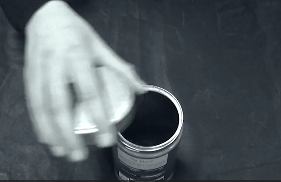 |
| Chiudere  (Close) | 100% | 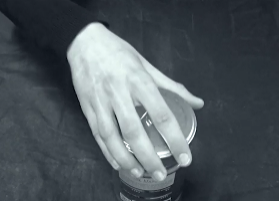 |
| Cucire  (Sew) | 92% | 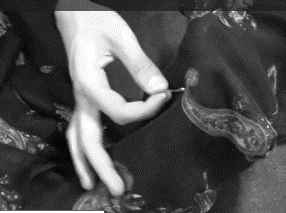 |
| Grattare  (Scratch) | 100% | 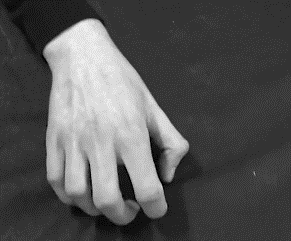 |
| Battere  (Beat) | 100% | 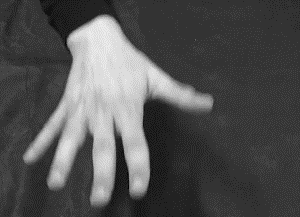 |
| Buttare  (Throw) | 100% | 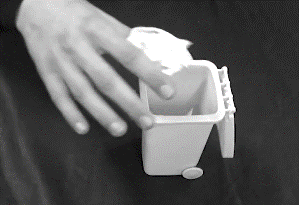 |
| Lavare  (Wash) | 91.7% | 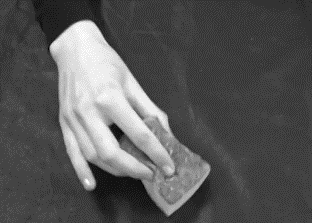 |
| Piantare  (Plant) | 92% | 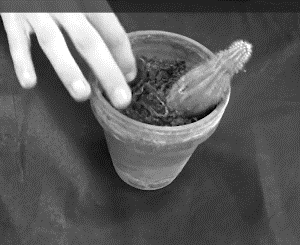 |
| Segnare  (mark) | 80% | 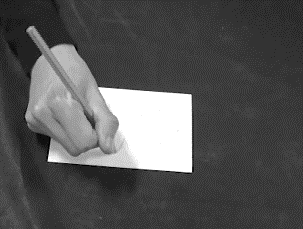 |
| Spargere  (Scatter) | 100% | 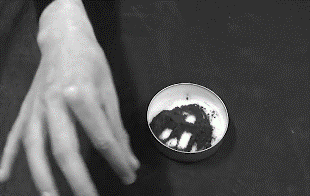 |
| Spezzare  (Break) | 86% | 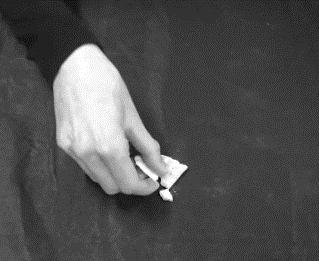 |
| Stringere  (Tighten) | 83,3 % | 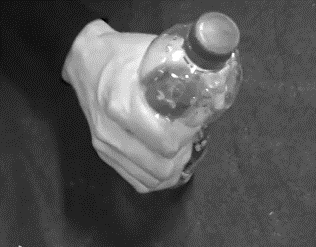 |
| Tagliare  (Cut) | 84% | 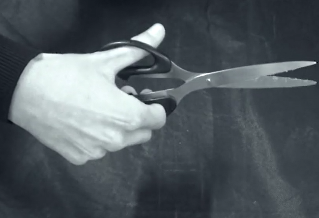 |
| Tendere  (Tend) | 85% | 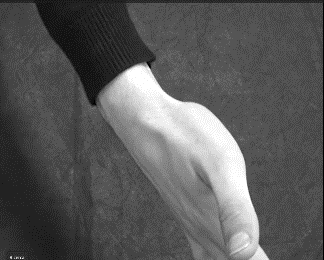 |
| Toccare  (Touch) | 100% | 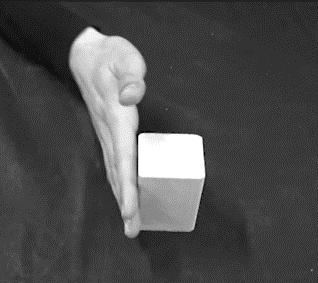 |
| Tirare  (Pull) | 100% | 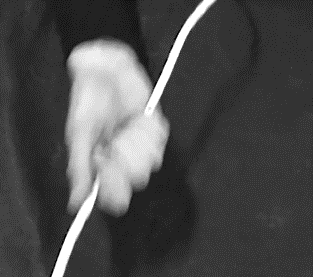 |
| Voltare  (Turn) | 92% | 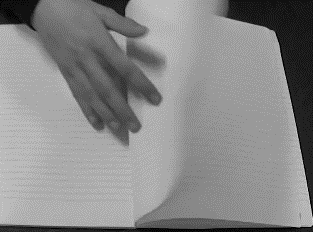 |

**2.2. Selected English videos.**

| **Action** | **Mean Accuracy** | **Frame taken from the video** |
| --- | --- | --- |
| Grasp | 100% | 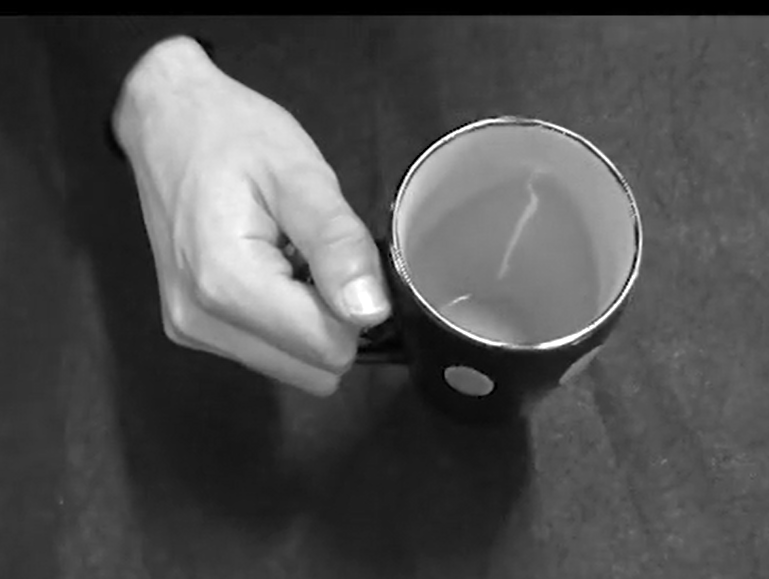 |
| Lift | 80% | 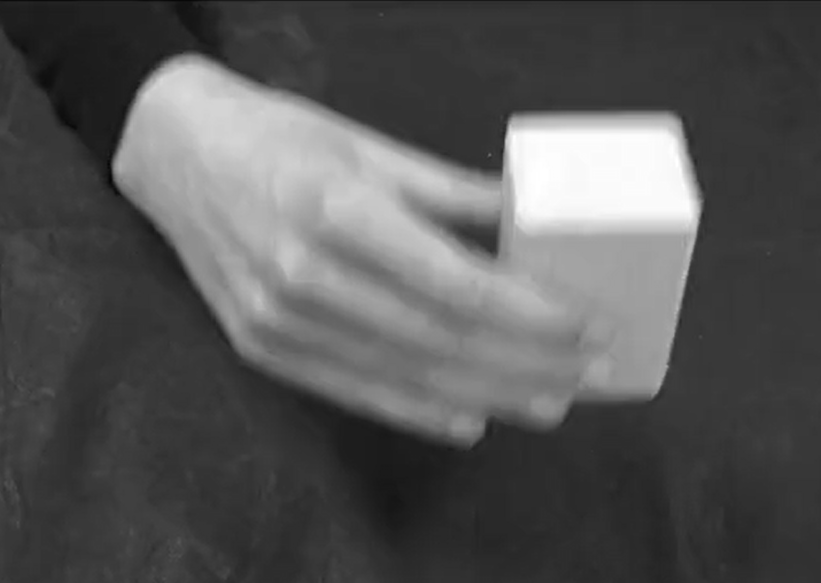 |
| Pick | 80% | 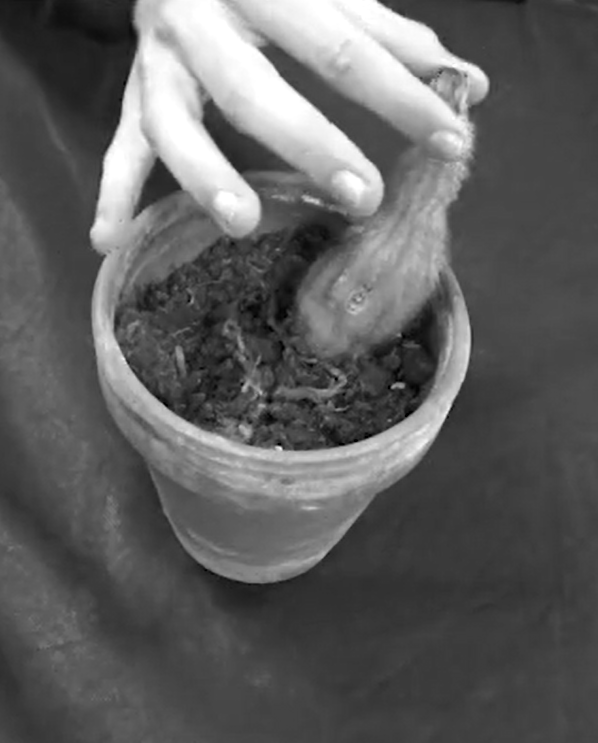 |
| Catch | 100% | 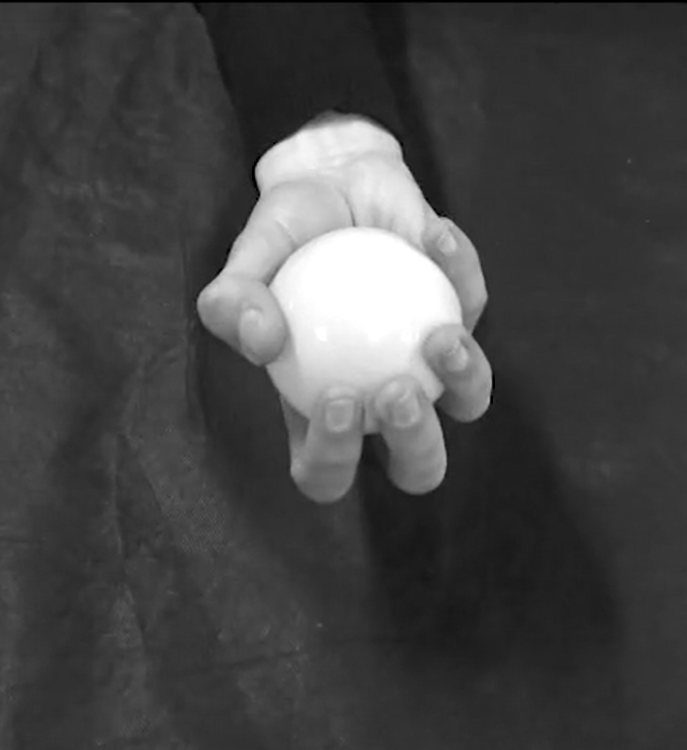 |
| Raise | 92% | 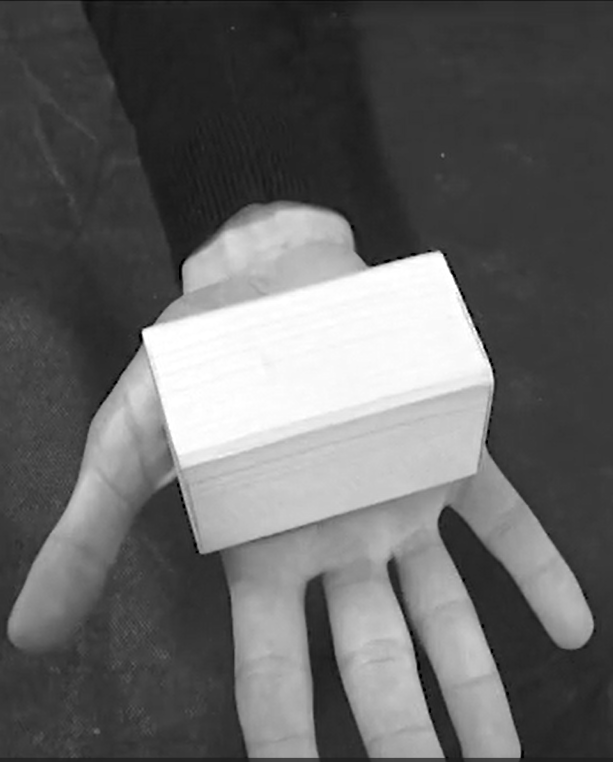 |
| Knock | 100% | 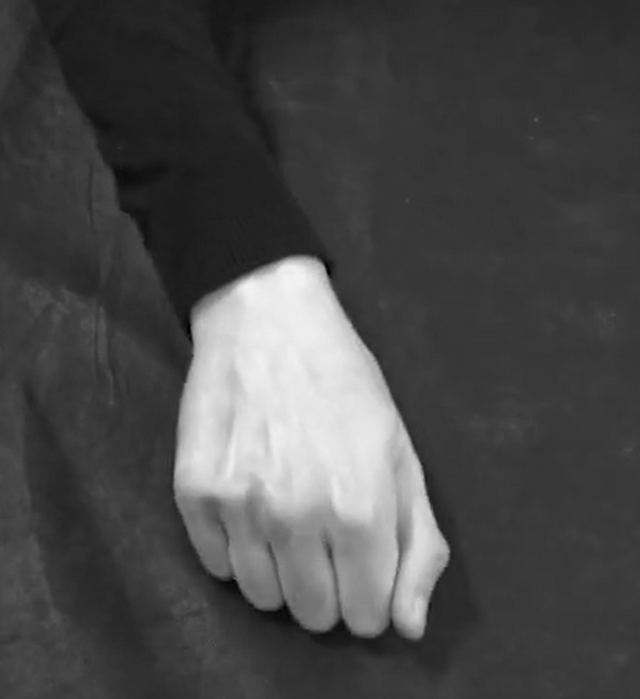 |
| Squeeze | 100% | 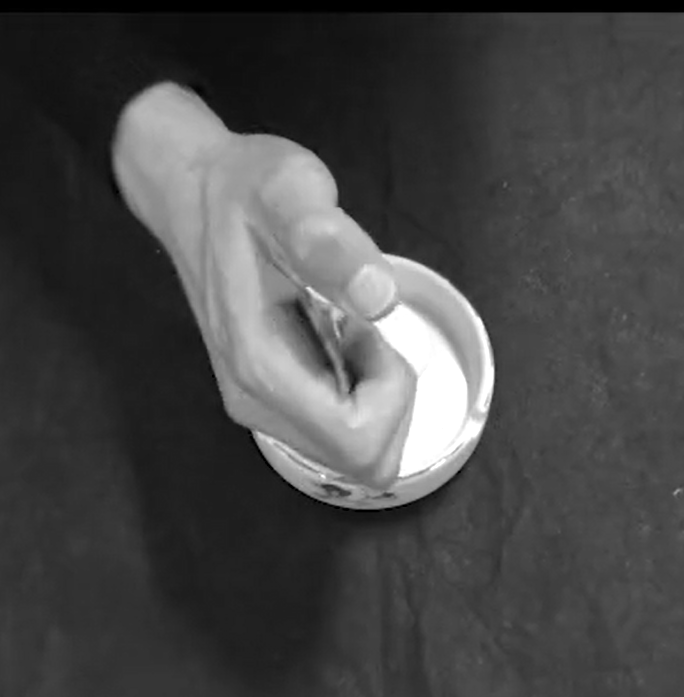 |
| Toss | 92% | 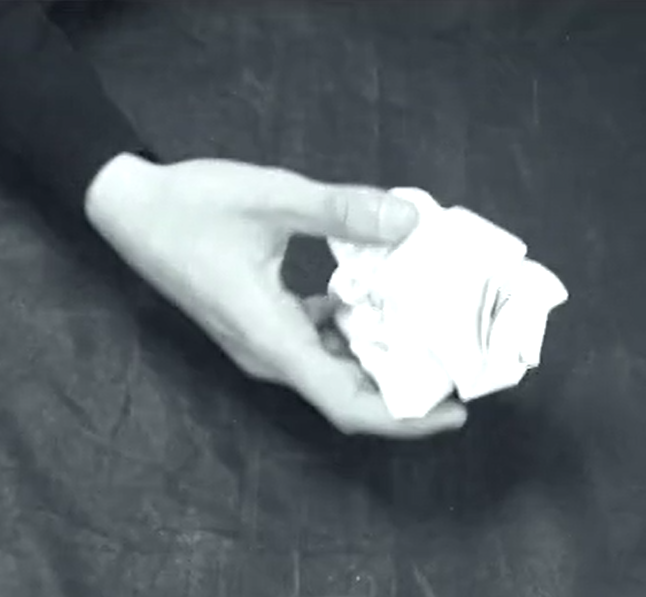 |
| Pour | 100% | 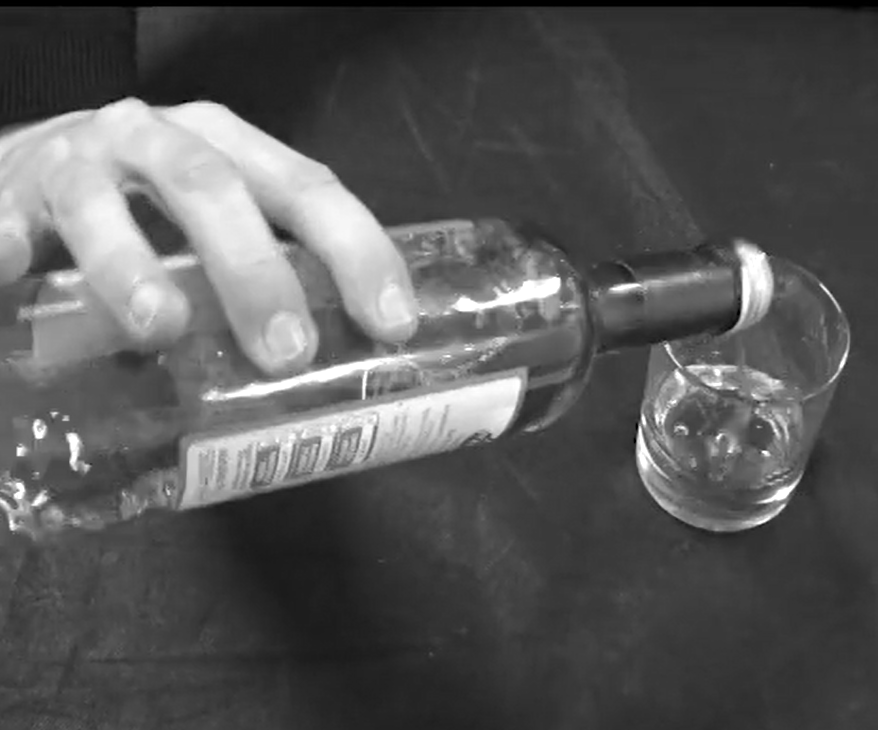 |
| Tear | 92% | 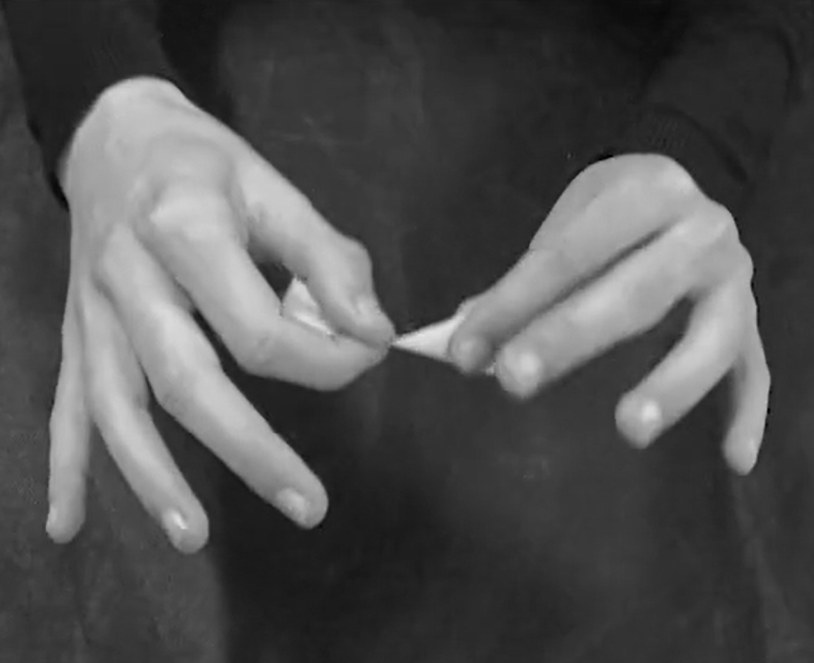 |
| Wipe | 80% | 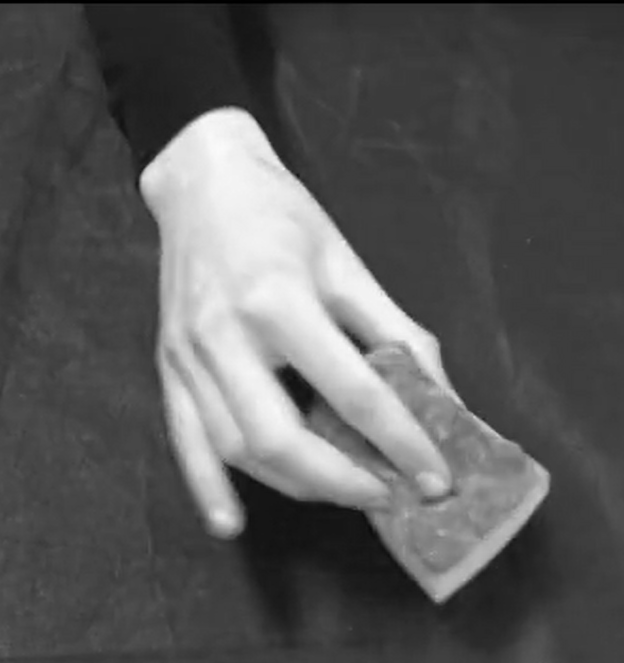 |
| Cut | 100% | 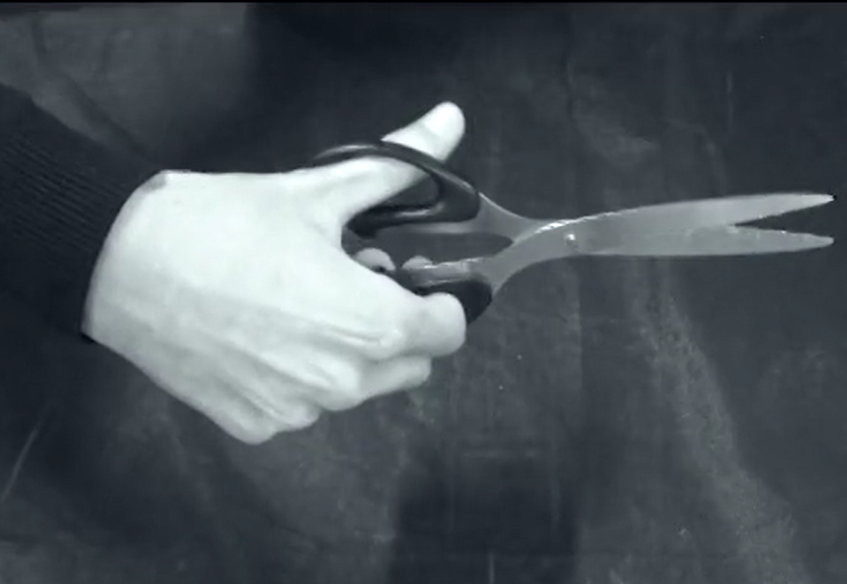 |
| Tighten | 90 % | 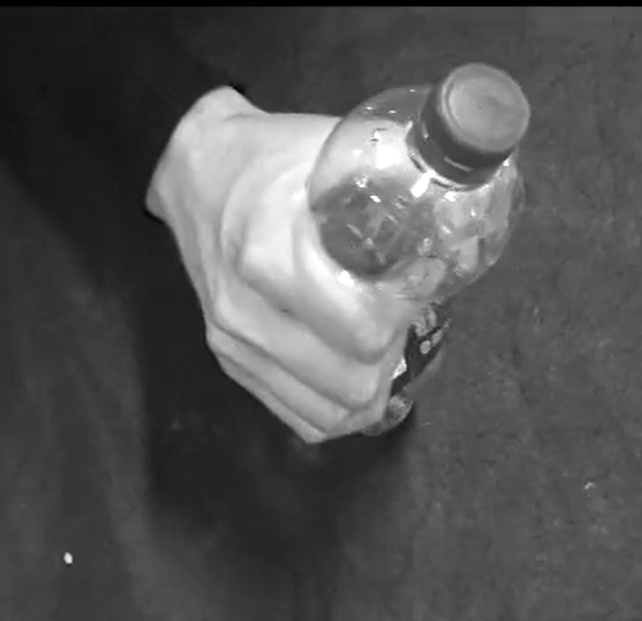 |
| Loosen | 96 % | 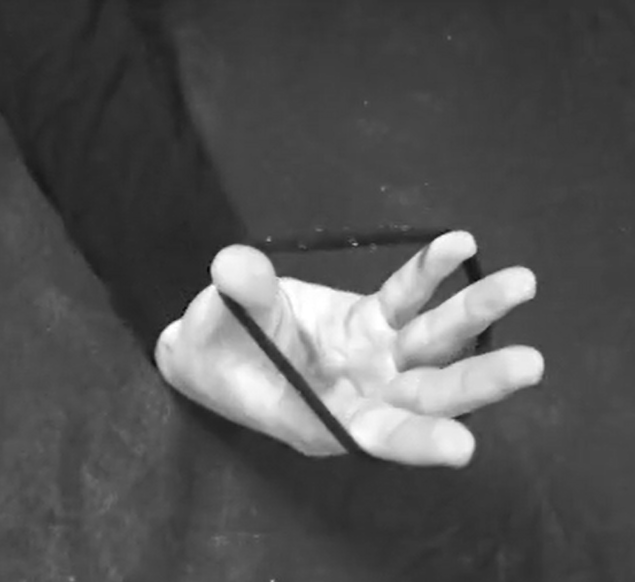 |
| Pull | 80% | 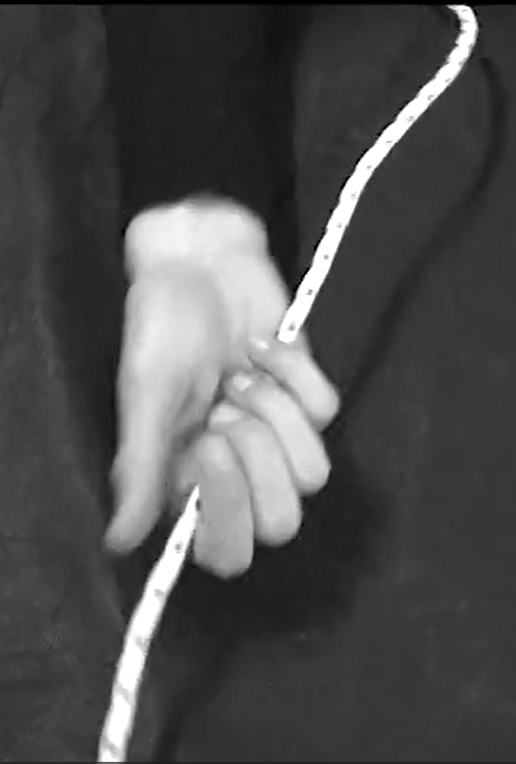 |
| Tickle | 100% | 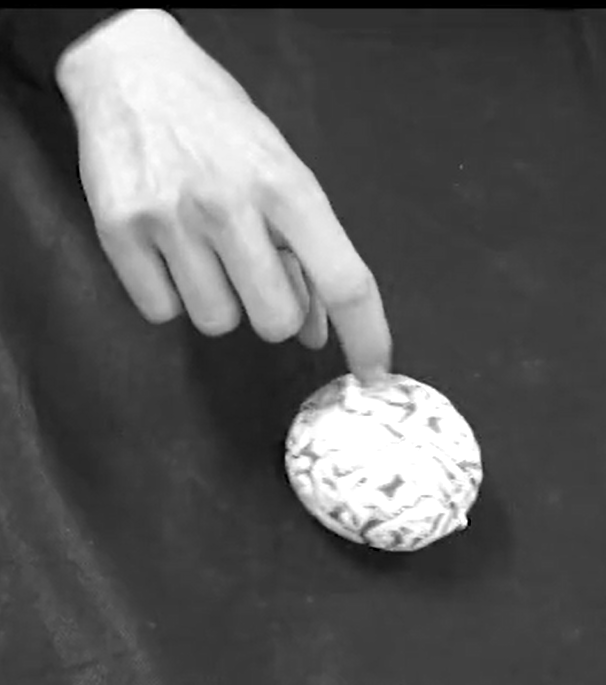 |
| Shake | 100% | 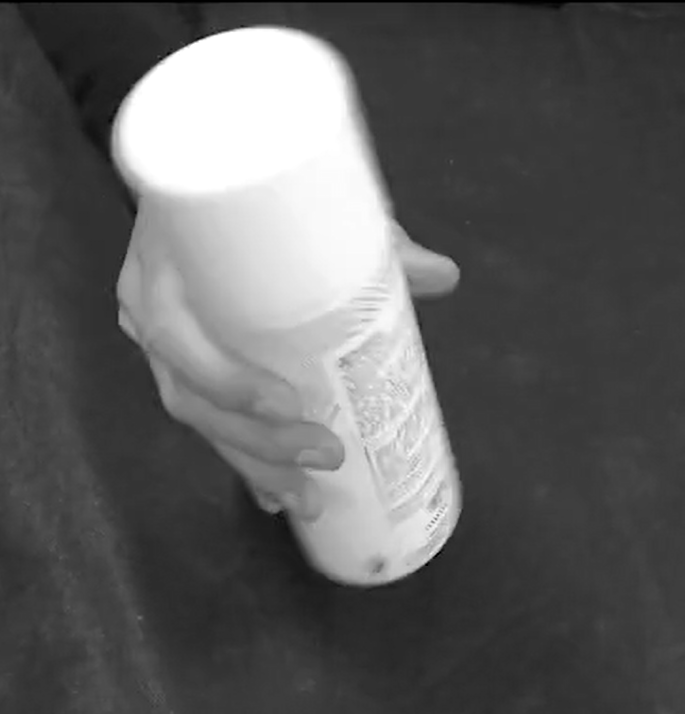 |
| Hammer | 100% | 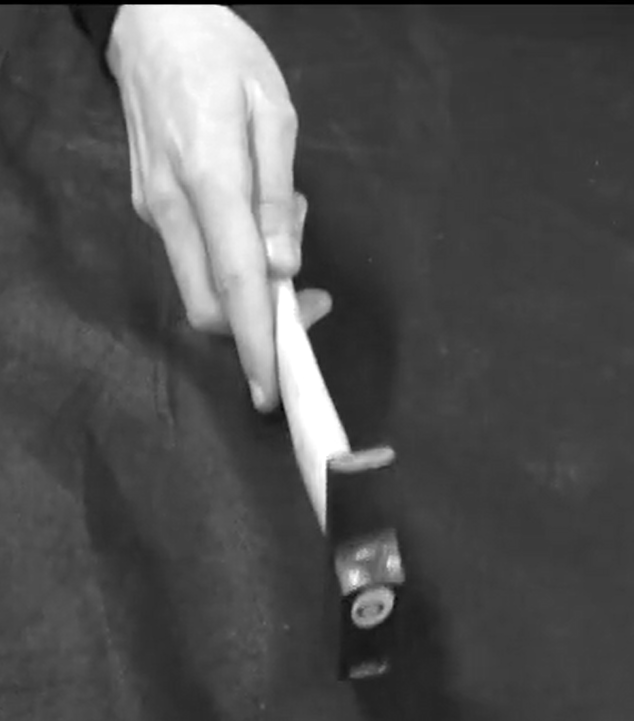 |

For further details on RTs and accuracy, visit the following link: [NET 2](https://univpr-my.sharepoint.com/:f:/g/personal/stefana_garello_unipr_it/EvwgHDV1bpNGrS-lovsn6fkBgJR78ssM7NmwnKJeQWZpgA?e=BQ5qIE)

**3. Data analysis and results**


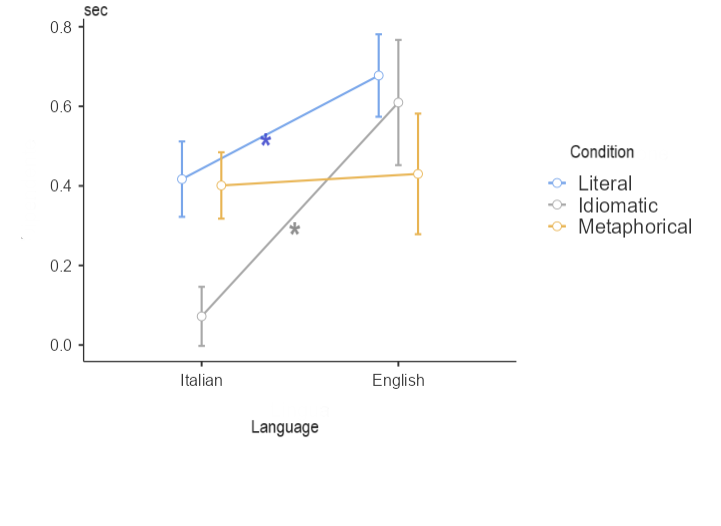


**Figure S1.** The graph shows how Δ value (unrelated – related modalities) change depending on the condition of the sentences, both in Italian and in English. The asterisk [*] indicates p < 0.05; only within language significant differences are showed.
